# Methane production from thermophilic co‐digestion of dairy manure and waste milk obtained from therapeutically treated cows

**DOI:** 10.1111/asj.12624

**Published:** 2016-05-12

**Authors:** Nilmini Beneragama, Masahiro Iwasaki, Kazutaka Umetsu

**Affiliations:** ^1^ Department of Environmental HygieneObihiro University of Agriculture and Veterinary MedicineObihiroJapan; ^2^ Agricultural Biotechnology Center, Faculty of AgricultureUniversity of PeradeniyaPeradeniyaSri Lanka

**Keywords:** *cefazolin‐resistant bacteria*, *co‐digestion*, *dairy manure*, *methane*, *waste milk*

## Abstract

Methane production from co‐digestion of dairy manure and waste milk, milk from cows treated with antibiotics for mastitis, was tested in a 2 × 4 factorial design. Four different waste milk percentages (w/w): 0% (SM), 10% (SMWM10), 20% (SMWM20) and 30% (SMWM30), were tested with two slurry percentages (w/w): 50% (A) and 25% (B) and the rest being manure at 55°C for 12 days in batch digesters. The results analyzed using a Gompertz model showed SMWM10 produced the highest methane production potential (P_m_)/g volatile solids added followed by SM in both A and B. This P_m_ of SMWM10 in A and B was statistically non‐significant (*P* > 0.05). More than 96% of cefazolin‐resistant bacteria and 100% of multi‐drug‐resistant bacteria reductions were observed in all the treatments. Inclusion of waste milk at 10% in single stage digester enhances the methane production from dairy manure and could offer added benefit of waste milk treatment and disposal.

## Introduction

Anaerobic digestion has gained continuous attention in treating organic wastes such as cow manure, since it produces biogas, a renewable energy source and a digestate that can be used as organic fertilizer. It is a complex biochemical process, in which organic compounds are mineralized to biogas, consisting primarily of methane and carbon dioxide, through a series of reactions mediated by several groups of microorganisms in an oxygen‐free environment. Although the production of biogas through anaerobic digestion offers significant advantages over other forms of waste treatment, biogas plants are difficult to run with economically profitable results if the process is based only on livestock manure. In this regard, co‐digestion strategies are of importance to enhance the methane production in agricultural biogas plants. In many countries, for instance, in Denmark (Raven & Gregersen 2007) and Germany (Weiland 2006), the digestion of manure and organic waste is a well‐established technological practice. This process consists of combining several wastes with complementary characteristics in order to improve methane production. The co‐digestion of cattle manure with municipal solid waste (Callaghan *et al*. 1999; Hartmann & Ahring 2005), food wastes (Neves *et al*. 2009), fruit and vegetable waste and chicken manure (Callaghan *et al*. 2002) and so on, has been shown to enhance methane production.

In many dairy farms, mastitis remains one of the most important and costly diseases which requires antibiotic therapeutic treatment leading to antibiotic residues in milk. The milk containing antibiotic residues should be withheld from use for a period recommended by the manufacturer and disposed of as a waste. Disposal of this waste milk into the environment leads to many adverse consequences, such as development of antibiotic‐resistant bacteria, contamination of surface water, ground water and so on. Milk is a highly polluting effluent, with a Chemical Oxygen Demand (COD) of around 190 000 mg/L, hence even small quantities that enter water courses can be highly polluting. It is therefore of considerable importance to facilitate proper treatment measures. In fact, the co‐digestion of waste milk from cows treated with antibiotics with dairy manure would be a better approach as it can provide additional benefits, in the form of reduced environmental pollution and increased gas production. However, it is obvious that the gas production may differ with the amount of waste milk incorporated into the system.

In the present study, co‐digestion of waste milk and dairy manure at different concentrations was investigated in batch experiments at thermophilic temperature (55°C) to determine: (i) the maximum concentration of milk which could be applied to a thermophilic digestion system without adversely affecting gas production; and (ii) in what extent the reduction of total culturable bacteria (TCB), cefazolin‐resistant bacteria (CRB) and multi‐drug‐resistant bacteria (MDRB) occurs during the 12 days period of digestion. The data from this experiment would be beneficial to determine the optimum amount of waste milk to be co‐digested with dairy manure for effective methane production and the apparent success of the treatment in eliminating CRB and MDRB.

## Materials and Methods

### Materials

Cow manure and digested slurry were obtained from a reception pit and digester of biogas plant at Obihiro University, Obihiro, Hokkaido, Japan, respectively. Cow manure, discharged to the pit, is obtained from a herd of lactating Holstein cows and collected daily from the concrete floor of a free stall barn. Digested slurry, the inoculum, is produced from digested cow manure in a digester operated at thermophilic temperature (55°C).

Cefazolin, a β‐lactam antibiotic, which suppresses the growth of bacteria by inhibiting cell wall synthesis (Kotra & Mobashery 1998) is frequently used in Obihiro University farm to treat cows with mastitis and the milk obtained from treated cows is withheld for a period recommended by the drug manufacturer and discarded. This waste milk was obtained and stored at 4°C for 5 days until used.

### Experimental design and procedure

Four waste milk percentages; 0, 10, 20 and 30 (w/w) were tested in co‐digestion with slurry and manure in two groups with 50% (A) and 25% (B) (w/w) slurry and the rest being the manure separately to have different organic loading rates. The four treatments in each group were tested in triplicate in 1 L batch digesters with an active volume of 700 mL at 55°C in a thermostatically controlled water bath for 12 days. For each treatment, slurry (S), manure (M) and waste milk (WM) were combined to produce desired ratios of slurry: manure : waste milk (for example, S 50%: M 50%, S 50%: M 40%: WM 10% etc.). The combined contents were thoroughly mixed with a hand mixer separately; 700 mL of each mixture was added to each digester in triplicate. Digesters were flushed with argon gas prior to sealing. Gas bags were fixed to each digester to collect the evolved biogas and the digesters were placed in a water bath at 55°C. Digestate samples were taken before and after the experiment to analyze for pH, TS (total solids), VS (volatile solids) degradation, volatile fatty acids (VFA) and population densities of total, CRB and MDRB.

### Culturing bacteria

Total, CRB and MDRB in slurry samples taken before and after the experiment were determined by plate counts on agar media. To culture bacteria, peptone, tryptone, yeast and glucose (PTYG) agar (a non‐selective medium) was prepared using 0.25 g of peptone, 0.25 g of tryptone, 0.5 g of yeast extract, 0.5 g of glucose, 0.03 g of MgSO_4_.7H_2_O, 0.0035 g of CaCl_2_.2H_2_O and 15 g of Bacto agar per liter to which cycloheximide (100 mg/L) was added as a fungicide (Kobashi *et al*. 2005). Agar media without any antibiotic was used to determine total bacteria, whereas agar media with cefazolin and a group of antibiotics (cefazolin, penicillin, oxytetracycline, vancomycin, kanamycin, streptomycin, neomycin, ampicillin, each at the concentration of 50 mg/L) were used to determine CRB and MDRB, respectively. Dilution plate method was used to determine population densities of all three groups of bacteria in all the samples. Samples were diluted by 10‐fold dilutions using phosphate‐buffered saline (PBS) (pH 7.4). The dilution plate method was conducted in three replicates and aliquots of 100 μL of sample were spread on the surfaces of three agar plates. The cultured plates were incubated at 30°C for 7 days. After incubation, the formed colonies were counted and calculated as colony‐forming units per gram of dry matter (cfu/g DM).

### Analytical methods

Total gas productions were monitored every other day. Wet gas meter was used to measure the volume of produced gas. All gas measurements were expressed at 0°C and a pressure of one atmosphere. Prior to measuring the volume of produced gas, gas composition of each gas bag was determined using gas chromatograph (GC) (Shimadzu GC‐14A, Houston, TX, USA) equipped with a thermal conductivity detector (stainless column and Porapak Q packing). The operational temperatures of injector port, column and the detector were 220, 150 and 220°C, respectively. Argon was the carrier gas at a flow rate of 50 mL/min.

TS and VS were measured according to the Standard Methods (2005). The pH was measured using a Horiba D‐55 pH meter. Slurry samples were analyzed for VFA (acetic, propionic, butyric and formic acids) with a high‐performance liquid chromatograph (HPLC, Shimadzu LC‐10 AD) with Shim‐Pack SCR‐102H column. The analytical procedure was described in detail by Kimura *et al*. (1994).

### Data analysis

Statistical analyses were performed using SAS version 9.2 (SAS Institute Inc., Cary, NC, USA). The effect of mixing ratios of digested slurry, dairy manure and waste milk to methane production in the anaerobic digester was analyzed using a Gompertz model as shown below.

(1) $$Mp\;=\;\mathrm{Pm}.\exp\left[-\exp\left\{\left(\frac{Rm}{\mathrm{Pm}}\right)\left(\lambda -t\right)e+1\right\}\right]$$

where Mp is the cumulative methane production (mL), P_m_ is the methane production potential (mL), R_m_ is methane production rate (mL/day), λ is lag‐phase time (day), e is exponential 1 and t is time.

All the parameters in the above equation were evaluated by performing regression with the solver to minimize the sum of the square errors (SSE) between the experiment and estimation. The goodness of the parameter fit was diagnosed by SSE and correlation coefficient (*R* ^2^).

## Results and Discussion

### Methane concentration and production

**Figure 1:**
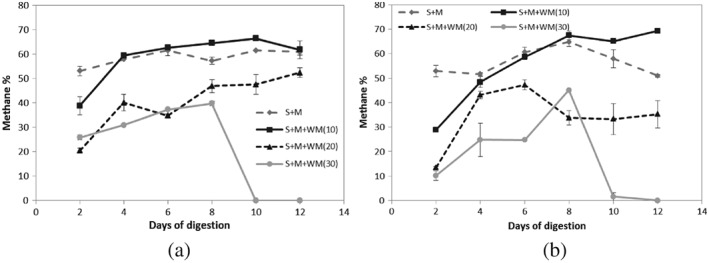
Methane concentration in produced gas from all mixed substrates (SM : SMWM10 : SMWM20 : SMWM30) of treatment A (50% slurry) [a] and B (25% slurry) [b] during the digestion at 55°C for 12 days. S, slurry; M, manure; WM, waste milk.

**Figure 2:**
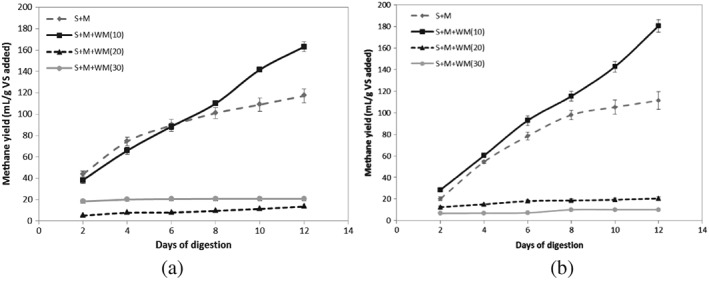
Methane yield based on g VS fed to the digesters of SM, SMWM10, SMWM20 and SMWM30 of treatment A (50% slurry) [a] and B (25% slurry) [b]. S, slurry; M, manure; WM, waste milk.

Methane concentrations in produced gas of treatments A and B over the digestion period are shown in Figure 1. At the second day of the digestion, the highest concentration of methane in produced biogas was obtained in the digesters without added waste milk (SM) in both A and B after which an increased methane concentration was observed in the digesters with 10% waste milk (SMWM10) in both A and B. The digesters added with 20% waste milk (SMWM20) and 30% waste milk (SMWM30) always produced the lower methane concentration than SM and SMWM10 despite the difference in slurry percentage. No methane production was observed in SMWM30 after day 10 in both situations. Figure 2 presents the cumulative methane volume produced /g VS added in each digester during the digestion period. Highest cumulative methane volumes of 163.05 mL and 180.43 mL were observed in both digesters of SMWM10 followed by SM producing 117.16 mL and 111.27 mL in treatments A and B, respectively. The methane volume produced /g VS added in SMWM10 was significantly higher (*P* < 0.05) than that of in SM in both treatments A and B. Similarly, methane production was significantly higher (*P* < 0.05) in SM than in SMWM20 and SMWM30 despite the different slurry percentages added. Digesters with SMWM20 and SMWM30 produced very low methane volumes as the methane concentrations of the produced gas were too low. Although an increasing methane volume with increasing waste milk percentage was expected, the results showed a different behavior. Inclusion of waste milk in high amounts would cause the inhibition of the process rather than increasing methane production.

Milk is known to contain significant quantities of butyric acid (Fessenden & Fessenden 1987) which is one of the VFAs produced by acidogenic bacteria during the anaerobic digestion process (Parkin & Owen 1986). Butyric acid is also formed during the anaerobic digestion of cattle manure from the fermentation of polysaccharide residues. This butyric acid is converted to acetic acid by a number of pathways. Bacteria which consume acetic acid produce about 70% of the methane generated by an anaerobic fermentation (Hobson *et al*. 1981). The introduction of substantial quantities of butyric acid into the system may explain the increasing volumes of methane produced by the digesters containing 10% waste milk. However, further increase of waste milk percentages to 20% and 30% caused a drastic reduction of methane level in the biogas. The observed reduction was probably caused by higher concentrations of total VFA with the addition of higher percentages of milk into the system. Argun *et al*. (2008) reported the accumulation of hydrogen and VFA can cause inhibition of the anaerobic degradation process. VFAs are a key intermediate in the process of anaerobic digestion and are also capable of inhibiting methanogenesis in high concentrations. The reason here is that the methanogens will not be able to metabolize the acetate produced by the acetogenic organisms until the number of methanogenic organisms has increased sufficiently. Waste milk may have hydrolyzed, rapidly releasing more VFA which probably reduced the pH to critical levels for methanogens, causing the system failure. Similar phenomenon has been observed by Lateef *et al*. (2012). Different manure: waste milk ratios were tested with different organic loading rates at 55°C to investigate bio‐hydrogen production and observed reduced methane concentration in produced gas with increasing the concentration of waste milk as a co‐substrate. Nevertheless, Callaghan *et al*. (1997) have reported that the addition of waste milk to a batch anaerobic digestion of cattle manure produced elevated methane production levels, with the highest methane production being observed in digesters receiving the highest loading of milk. This observation also tallied with the current experiment as the maximum waste milk percentage that has been included into the digesters under shock loading conditions was ca. 15%. Waste milk at 15% was not tested in the current study. It can be seen from the results that inclusion of 10% waste milk into the system generated the highest methane volume and 20% and more produced trace volumes. Therefore, it can be expected that 15% of waste milk in the system would also produce higher methane volumes.

The digesters receiving waste milk showed a rapid increase in carbon dioxide production in the first 2 days of digestion, after which the production decreased (data not shown). This increase of carbon dioxide was more prevalent in the digesters which received more waste milk as a percentage (20% and 30%). This can be explained by the presence of butyric acid in milk. The conversion of butyric acid to acetic acid causes the observed rise in carbon dioxide concentration of the biogas (Callaghan *et al*. 1997). However, increasing methane concentrations were observed in all the digesters after 2 days with highest values observing in SM and SMWM10 of both treatments. The reason here is that the methanogens would not be able to metabolize the acetate produced by the acetogenic organisms until the methanogenic organisms have increased to a number to cope with the increased levels of substrate. The accumulation of VFA caused by high organic loading rates in SMWM20 (72.9 g VS/L) and SMWM30 (74.5 g VS/L) led to the digester failure.

### Estimation of methane production potential using a Gompertz model

The Gompertz model provided in Eq. (1) was applied to fit the methane production profiles. The cumulative methane production curves from the two different treatments, each with four mixing ratios, were well described with Eq. (1). Each variable of the model was calculated with different mixing ratios of two treatments for methane production potential/g VS added. Table 1 shows the model parameters identified from regression of the methane production profiles.

**Table 1:** Gompertz model parameters identified from regression of the methane production profiles at different mixing ratios of slurry (S) (produced from digested cow manure in a digester operated at thermophilic temperature: 55°C), manure (M) and waste milk (WM) (obtained from a herd of lactating Holstein cows) of treatments A (with 50% slurry) and B (with 25% slurry)

Treatment	S% (w/w)	M% (w/w)	WM% (w/w)	P_m_ (mL CH_4_/ g VS added)	R_m_ (mL/day)	λ (days)	*R* ^2^
SM(A)	50	50	0	112.66	18.98	0	0.9935
SMWM(10)	50	40	10	196.15	16.17	0	0.9998
SMWM(20)	50	30	20	12.71	1.65	0	0.9995
SMWM(30)	50	20	30	20.67	11.83	0	0.8171
SM(B)	25	75	0	114.50	16.33	0.77	0.9994
SMWM(10)	25	65	10	298.12	15.71	0.49	0.9992
SMWM(20)	25	55	20	19.02	5.67	0	0.9218
SMWM(30)	25	45	30	9.23	2.82	0	0.917

P_m_, methane production potential; VS, volatile solids; R_m_, methane production rate; λ, lag phase time; *R* ^2^, regression co‐efficient)

In treatment A, the maximum methane production potential (P_m_) values of SM, SMWM20 and SMWM30 were found to be almost similar to the values observed. However, the P_m_ value of SMWM10 was found to be higher than the observed value. The mixing ratios of treatment B followed the similar pattern with a higher P_m_ value in SMWM10. This reveals that in both cases, the digestion in SMWM10 was not completed within 12 days and the cumulative methane production curve (Fig. 2) of SMWM10 clearly indicates the continuing methane production. The digester with SMWM10 showed the highest P_m_/g VS added followed by SM in both A and B. This P_m_ of SMWM10 in A and B was statistically non‐significant (*P* > 0.05) and the same could be observed with SM. Nevertheless, in each treatment, SMWM10 showed a significantly higher (*P* < 0.05) P_m_ than SM. This further demonstrates that co‐digestion of dairy manure with 10% waste milk would enhance methane production and incorporation of more waste milk (>20%) would severely affect the methane production process in single‐stage anaerobic digestion.

Each mixing ratio between treatments A and B showed no significant difference in methane yields, although the highest methane yield observed in SMWM10 in each treatment showed a significant difference with respective SM. The methane production rates (mL/day) of SM and SMWM10 in treatment A were almost similar to the respective values in treatment B (Table 1) as calculated by Gompertz equation. However, methane production in both SM and SMWM10 of treatment B exhibited a lag phase time (λ) in the first day of digestion. Digesters added with 50% slurry as the inoculum showed no λ. SM and SMWM10 added with 25% slurry showed a λ of 0.77 days and 0.49 days respectively. Thereafter methane production steadily increased, reaching a final methane volume of 111.27 mL/g VS added and 180.43 mL/g VS added, respectively, showing incomplete digestion at the end of 12 days. The methane yield was expected to keep increasing and eventually reach a value of 114.5 mL/g VS added and 298.12 mL/g VS added in SM and SMWM10, respectively.

The observed lag phase in SM and SMWM10 in treatment B might be due to higher organic loading rate and higher temperature resulting in faster biodegradation of organic matter and accumulation of VFA in the digester. Consequently, the methanogenic population was expected to take some time to recover. Since the initial volatile solid percentages of those digesters were little higher than the digesters in treatment A, it showed a λ of less than a day. Moreover, the observed low λ in SMWM10 than SM can be attributed to the presence of waste milk, which is easily degradable, in SMWM10 and subsequently releasing VFA into the system, facilitating methane production by methanogens.

### VS degradation and VFA production

VS degradation percentage and characteristics of each mixing ratio (slurry : manure : waste milk) of treatments A and B are presented in Table 2. The maximum VS degradation was achieved with SMWM30 in both A (28.37%) and B (24.14%). Although an increasing VS degradation percentage was expected with increased portions of waste milk in the mixtures, in both treatments the results did not show any relationship between percentage VS degraded and waste milk percentage added. This is likely due to the different organic loading rates in each mixture.

**Table 2:** Volatile solids (VS) degradation and changes in volatile fatty acids (VFAs) and pH of slurry (S), manure (M), waste milk (WM) substrates of treatment A (with 50% slurry) and B (with 25% slurry)

Treatment	Digester	VS degradation (%)	Total VFA initial (g/L)	Total VFA final (g/L)	pH initial	pH final
A (50% slurry)	SM	19.16 ± 2.83†	3.64 ± 0	0.4 ± 0.06	7.24 ± 0.00	7.81 ± 0.02
	SMWM10	22.91 ± 3.24	5.10 ± 0.68	2.93 ± 0.07	7.42 ± 0.00	7.79 ± 0.01
	SMWM20	16.24 ± 2.5	4.50 ± 0.07	11.21 ± 0.42	7.49 ± 0.00	7.11 ± 0.07
	SMWM30	28.37 ± 1.72	3.35 ± 0.34	15.28 ± 0.02	7.61 ± 0.00	6.22 ± 0.02
B (25% slurry)	SM	27.55 ± 1.46	5.23 ± 0.00	1.58 ± 0.16	7.34 ± 0.00	8.14 ± 0.04
	SMWM10	22.71 ± 1.32	6.41 ± 0.09	3.37 ± 0.31	7.37 ± 0.00	8.16 ± 0.03
	SMWM20	18.59 ± 3.05	4.59 ± 0.42	14.22 ± 0.44	7.45 ± 0.00	6.74 ± 0.10
	SMWM30	24.14 ± 1.4	5.73 ± 0.11	17.15 ± 0.12	7.61 ± 0.00	5.94 ± 0.01

† Data in table are means ± standard errors.

**Figure 3:**
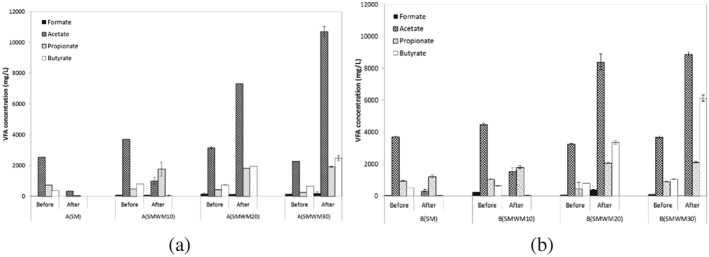
Volatile fatty acid (VFA) concentration of mixed substrates (SM : SMWM10 : SMWM20 : SMWM30) at two different slurry percentages, 50% slurry (a) and 25% slurry (b), before and after the digestion. Values are means with standard error. S, slurry; M, manure; WM, waste milk.

The initial and final VFA concentrations of all mixing ratios of treatments A and B are shown in Figure 3. The initial total VFA concentrations of all four mixing ratios were higher in treatment B than the respective mixing ratios of treatment A. Incorporation of 25% slurry in treatment B ended up with more manure percentage in all the mixtures. This might have led to higher total VFA in the mixtures of treatment B. In both cases, SMWM10 showed the highest initial total VFA concentration (Table 2). The differences in mean VFA productions between SM, SMWM10, SMWM20 and SMWM30 of treatment A were significant (*P* < 0.005). Similarly, the productions differed (*P* < 0.005, *P* < 0.05) between all mixing ratios of treatment B. Acetic acid was present in highest concentrations in all the digesters of A and B before the digestion. After 12 days of digestion at 55°C, both SM and SMWM10 of treatment A and B ended up with very low total VFA values. However, very high total VFA values observed in SMWM20 and SMWM30 of both treatments A and B at the end of the digestion demonstrated an accumulation of VFA which subsequently affected the process stability. Many researches have shown the inhibition of anaerobic digestion due to the accumulation of VFA. Siegert and Banks (2005) have shown that fermentation of glucose is inhibited at total VFA concentrations above 4 g/L. The total final VFA values of SMWM20 (A and B) and SMWM30 (A and B) of the current study are excessively high, as can be seen in Table 2. This coincided well with the low gas production in those digesters and the inhibition of methanogenesis after 3 to 4 days.

Acetate, propionate and butyrate were the most prevalent VFA in all the mixing ratios of treatments A and B (Fig. 3). High productions of butyrate were observed in mixing ratios with more waste milk (SMWM20 and SMWM30) in both treatments. Reduced total VFA concentration in SM and SMWM10 in A and B at the end of the digestion period would explain the use of VFA by methanogenic bacteria for methane generation and it was further confirmed by the observed methane production in those digesters (Figs 1 and 2). Acetic acid is usually present in higher concentrations than other fatty acids during anaerobic digestion (Wang *et al*. 1999). The same could be observed in the current study. The observed higher total VFA concentration in SMWM10 than that in SM of both treatments at the end of 12 days would indicate the incomplete digestion in SMWM10 and increasing cumulative methane production in SMWM10 was observed (Fig. 2). As predicted by Gompertz equation (1), the methane yield of SMWM10 of treatments A and B were expected to increase and reach a value of 196.15 mL/g VS added and 298.12 mL/g VS added, respectively. Increase in fatty acids with increasing milk percentage (20% and 30%) can be indicative of an overload of the organic loading rate and subsequent inhibition of the process. Excessive fatty acids are considered an inhibitor of methanogenesis and toxic only in their non‐ionized forms. The relative proportion of the ionized and non‐ionized forms is pH dependent. Volatile fatty acids are toxic below pH 7 (Mata‐Alvarez 2002).

### pH

**Figure 4:**
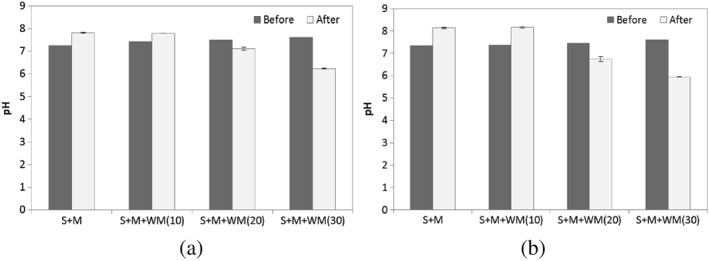
pH change of mixed substrates (SM : SMWM10 : SMWM20 : SMWM30) of treatment A (50% slurry) [a] and B (25% slurry) [b] during digestion at 55°C for 12 days. S, slurry; M, manure; WM, waste milk.

pH is a good indicator of anaerobic digester stability. pH changes of mixed substrates of treatments A and B during the digestion period are shown in Figure 4. The pH of all mixing ratios of both treatments measured at the beginning of the experiment revealed an increase of pH with increasing milk percentage added. Therefore, digesters added with 30% milk showed the highest pH in both A and B treatments. However, in all the digesters pH remained in the range 7.24‐7.61 which is suitable for the anaerobic digestion process. The ideal pH range for anaerobic digestion is very narrow: pH 6.8‐7.2. The growth rate of methanogens is greatly reduced below pH 6.6 (Mosey & Fernandes 1989), whereas an excessively alkaline pH can lead to disintegration of microbial granules and subsequent failure of the process (Sandberg & Ahring 1992). At day 12 after the digestion, alkaline pH values in SM and SMWM10 and acidic pH values in SMWM20 and SMWM30 in both cases except more neutral pH in SMWM20 of treatment A (Fig. 4) could be observed. Lowest pH values of 6.22 and 5.94 were observed in SMWM30 of treatments A and B, respectively. The lower pH values observed in SMWM20 and SMWM30 would be due to the accumulation of VFA in the systems. Buffer capacity is often referred to as alkalinity in anaerobic digestion. Buffer capacity, which is reduced by accumulation of short chain fatty acids significantly before pH decreases, can be best accomplished by reducing the organic loading rate or direct bicarbonate addition (Guwy *et al*. 1997). This suggests that the inhibition caused by increasing milk percentage in the inoculum‐to‐feed ratio might be counteractive with the addition of bicarbonate to the system. However, further research is needed in that aspect.

### Total, CRB and MDRB

**Figure 5:**
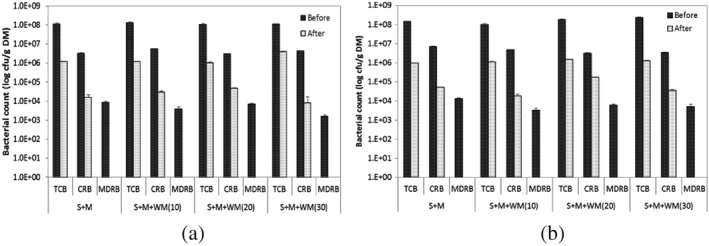
Bacterial load (TCB, CRB, MDRB) of mixed substrates (SM : SMWM10 : SMWM20 : SMWM30) of treatment A (50% slurry) [a] and B (25% slurry) [b], before and after the digestion at 55°C for 12 days. Values are means with standard error. S, slurry; M, manure; WM, waste milk. TCB, total culturable bacteria; CRB, cefazolin‐resistant bacteria; MDRB, multi‐drug‐resistant bacteria.

Bacterial loads of mixed substrates of treatments A and B are presented in Figure 5. The overall reductions as a percentage of initial concentration of TCB, CRB and MDRB of each mixing ratio of treatments A and B are shown in Table 3. TCB reduction as a percentage of initial concentration of 96.5‐99.0% and 98.8–99.4% were observed in all mixing ratios of treatments A and B, respectively, at the end of the experiment. The values differed between 98.4–99.8% and 98.8–99.4% for TCB, respectively. MDRB showed a 100% reduction in all the digesters. In treatment A, TCB reduction as a percentage of initial concentration of SMWM30 differed significantly (*P* < 0.05) from other mixing ratios, whereas SMWM20 showed a significant difference (*P* < 0.01) for CRB. All mixing ratios of treatment B did not follow the similar differences of reduction percentages. The values for TCB of SMWM10 differed significantly (*P* < 0.05) from other mixing ratios. Conversely, SMWM20 and SMWM30 showed significant difference (*P* < 0.005) with other mixing ratios for CRB reduction. No significant difference (*P* > 0.05) was observed between each mixing ratio of treatments A and B, revealing that the two different slurry percentages added would not cause any effect on reduction of TCB, CRB or MDRB. Moreover, increasing waste milk percentage in the mixture did not show any association with the level of reduction of TCB, CRB or MDRB. These findings were supported by the findings of Lateef *et al*. (2012) that the overall reduction of CRB as a percentage of initial concentration was dependent upon manure : waste milk and organic loading during the 5 day period of bio‐hydrogen production. It has been shown that the reduction was significantly higher (*P* < 0.05) at high organic loading (40 g VS/L and 60 g VS/L) than at low organic loading (20 g VS/L), whereas increasing the organic loading above 40 g VS/L was associated with non‐significant (*P* > 0.05) increase in the level of reduction. In the current experiment, the organic loading of all the mixing ratios of both treatments lies between 62.3 g VS/L and 74.5 g VS/L, which are considered as high organic loading rates.

**Table 3:** Overall bacterial reduction as a percentage of initial concentration of mixed substrates (different mixing ratios of slurry (S), manure (M) and waste milk (WM)) of treatments A (with 50% slurry) and B (with 25% slurry)

Treatment	Digester	TCB (%)	CRB (%)	MDRB (%)
A	SM	98.92 ± 0.17†	99.51 ± 0.20	100 ± 0.00
	SMWM10	99.04 ± 0.17	99.48 ± 0.07	100 ± 0.00
	SMWM20	99.05 ± 0.08	98.42 ± 0.08	100 ± 0.00
	SMWM30	96.51 ± 0.31	99.80 ± 0.20	100 ± 0.00
B	SM	99.32 ± 0.05	99.28 ± 0.04	100 ± 0.00
	SMWM10	98.86 ± 0.10	99.62 ± 0.09	100 ± 0.00
	SMWM20	99.21 ± 0.07	94.51 ± 0.25	100 ± 0.00
	SMWM30	99.41 ± 0.09	98.95 ± 0.12	100 ± 0.00

TCB, total culturable bacteria; CRB, cefazolin‐resistant bacteria; MDRB, multi‐drug‐resistant bacteria)

† Data in table are means ± standard errors.

The observed reduction of MDRB was expected as the initial MDRB count was far below the values of TCB and CRB. The initial bacterial count affects the order of magnitude of bacterial reduction. Temperature together with suitable exposure time are considered to be the most important factors for microbial growth inhibition in an aerobic digestion environment (Sahlstrom 2003). Thermophilic temperature causes greater reduction of antibiotic‐resistant bacteria than mesophilic temperature (Ghosh *et al*. 2009). Limited information is available on how anaerobic digestion technology and its associated operating conditions affect the quantities of antibiotic‐resistant bacteria (Diehl & Lapara 2010). VFA concentration alone, or in combinations with pH, temperature, exposure time and the degree of sensitivity of specific types of microorganisms, is believed to impact upon injury of microorganisms in anaerobic digestion (Abdul & Lloyd 1985; Salsali *et al*. 2008).

The study clearly shows that co‐digestion of dairy manure and waste milk from mastitic cows treated with antibiotics is plausible, aiming to produce increased amounts of biogas and to reduce antibiotic‐resistant bacteria in substantial amounts. In fact, anaerobic co‐digestion would be an ideal candidate for treatment and disposal of waste milk from mastitic cows. However, the net production of VFA increased with increased percentage of waste milk in the mixture, resulting in more acidic final pH and eventually leading to digester failure. The most suitable waste milk concentration to be combined with dairy manure for anaerobic co‐digestion was found to be 10% of total mixture regardless of the slurry percentage added. Although there was no significant difference in gas production between the digesters added with 50% and 25% slurry, digesters with lower slurry percentage as an inoculum needed some lag phase for the digestion to be started. Given the rate of production of waste milk, it would be feasible to incorporate 10% waste milk into the system. In situations where there is high waste milk production, the best approach would be to perform the digestion in two stages (acedogenesis and methanogenesis). The VFA produced in the acedogenesis (first) stage can be fed to the second digester for methanogensis to occur. Here the first stage serves as a pretreatment for the second stage and it would be a remedy for methane inhibition due to overloading and subsequent accumulation of VFA in a single‐stage digester.

### Conclusions

This study showed that inclusion of waste milk as a co‐substrate in methane production from dairy manure could improve methane yield. Optimal waste milk percentage to be added in both 50% (w/w) and 25% (w/w) slurry conditions was shown to be 10% (w/w). Higher waste milk percentages tested (20% and 30%) caused digester failure due to accumulation of VFA and subsequent pH drop. The study also showed that all treatments were very effective in controlling CRB and MDRB. Therefore, thermophilic anaerobic co‐digestion would be an ideal treatment technology to treat waste milk from antibiotic‐treated mastitic cows.
